# Support group for long term suffers of anorexia

**DOI:** 10.1186/2050-2974-1-S1-O7

**Published:** 2013-11-14

**Authors:** Heather Litchfield, Harry Derham

**Affiliations:** 1 Eating Disorders Inpatient Unit, NorthWestern Mental Health, Royal Melbourne Hospital, Australia

## 

At least 10% of people living with Anorexia will develop a chronic course of the illness, there has been very little research or literature surrounding this patient group. The Eating Disorder Program at the Royal Melbourne Hospital has commenced a new initiative for clients with severe and enduring eating disorders. This group aims to engage these patients from a different stand point, operating on a recovery focus, aiming to improve the quality of life, as it aims to maximize the individual's potential despite the illness.

The group is primarily supportive and mostly patient driven, topics discussed within the group are brought up by participants. It is facilitated by a consultant of the outpatient team and social worker of the inpatient unit. The group will remain an open group with a maximum of 9 participants running once a month, with the aim for the group to become self supporting in the long term.

Most of the participants assessments show all reasonable attempts to reach full recovery have been made, many are recurrently admitted or who have maintained a low weight for a long period of time have lost social skills and lead lonely lives. This group aims to improve self-esteem and promote friendship groups within the patient group.

This abstract was presented in the **Adult Treatment and Services** stream of the 2013 ANZAED Conference.

